# Cyanotic Congenital Heart Disease The Coronary Arterial Circulation

**DOI:** 10.2174/157340312801215836

**Published:** 2012-02

**Authors:** Joseph K Perloff

**Affiliations:** The Ahmanson/UCLA Adult Congenital Heart Disease Center, David Geffen School of Medicine at UCLA, Los Angeles, California, USA

**Keywords:** Coronary arteries, cyanosis, congenital heart disease.

## Abstract

**Background:**

The coronary circulation in cyanotic congenital heart disease (CCHD) includes the extramural coronary arteries, basal coronary blood flow, flow reserve, the coronary microcirculation, and coronary atherogenesis.

**Methods:**

Coronary arteriograms were analyzed in 59 adults with CCHD. Dilated extramural coronaries were examined histologically in six patients. Basal coronary blood flow was determined with N-13 positron emission tomography in 14 patients and in 10 controls. Hyperemic flow was induced by intravenous dipyridamole pharmacologic stress. Immunostaining against SM alpha-actin permitted microcirculatory morphometric analysis. Non-fasting total cholesterols were retrieved in 279 patients divided into four groups: Group A---143 cyanotic unoperated, Group B---47 rendered acyanotic by reparative surgery, Group C---41 acyanotic unoperated, Group D---48 acyanotic before and after operation.

**Results:**

Extramural coronary arteries were mildly or moderately dilated to ectatic in 49/59 angiograms. Histologic examination disclosed loss of medial smooth muscle, increased medial collagen, and duplication of internal elastic lamina. Basal coronary flow was appreciably increased. Hyperemic flow was comparable to controls. Remodeling of the microcirculation was based upon coronary arteriolar length, volume and surface densities. Coronary atherosclerosis was absent in both the arteriograms and the necropsy specimens.

**Conclusions:**

Extramural coronary arteries in CCHD dilate in response to endothelial vasodilator substances supplemented by mural attenuation caused by medial abnormalities. Basal coronary flow was appreciably increased, but hyperemic flow was normal. Remodeling of the microcirculation was responsible for preservation of flow reserve. The coronaries were atheroma-free because of the salutory effects of hypocholesterolemia, hypoxemia, upregulated nitric oxide, low platelet counts, and hyperbilirubinrmia.

## GENERAL BACKGROUND

The data included herein are based upon five published research studies that dealt with the extramural coronary arteries, [1,2] basal coronary blood flow and flow reserve, [3] the coronary microcirculation [4] and coronary atherogenesis. [5] 

Dilated tortuous extramural coronary arteries in CCHD was first mentioned in the literature as an incidental photograph published in 1955 without comment. [6] In 1966, Bjork reported ectasia of the coronary arteries in a cyanotic adult, [7] and two years later, Bjork’s observation was confirmed by angiography and at necropsy [1]. In 1971, dilatation and tortuosity of extramural coronaries were described in adults acclimatized to the rarefied atmosphere of the Peruvian Andes, indicating that the coronary abnormalities were not congenital, but instead were responses to high altitude [8].

### The Extramural Coronary Arteries

**Fig. (1):**
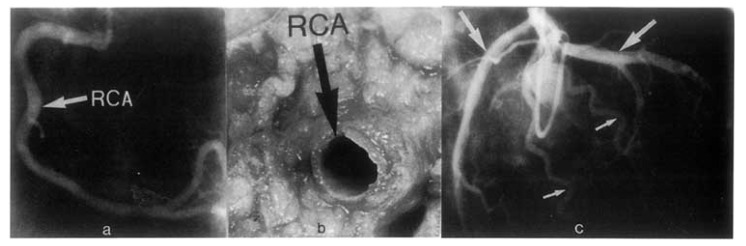
**a**) Coronary arteriogram illustrating moderate dilatation of the right coronary artery (RCA) in a 46 year old cyanotic male with Eisenmenger syndrome. **b**) Necropsy specimen illustrating moderate dilatation of the RCA in a 38 year old cyanotic male with Eisenmenger syndrome. **c**) Coronary arteriogram from a 53 year old cyanotic male with Eisenmenger syndrome. The circumflex and anterior descending arteries (larger upper arrows) are moderately dilated; the diagonal arteries (smaller lower arrows) are moderately dilated and tortuous. Coronary arterial size (diameter) was judged by comparison with the diameter of standard angiographic catheters. (From Chugh R, Perloff JK *et al.* Am J Cardiol 2004; 94: 1355-57).

Extramural coronaries were mildly dilated, moderately dilated or ectatic and tortuous in 83% (49/59) of the coronary arteriograms [2] (Fig. **1**).

**Fig. (2):**
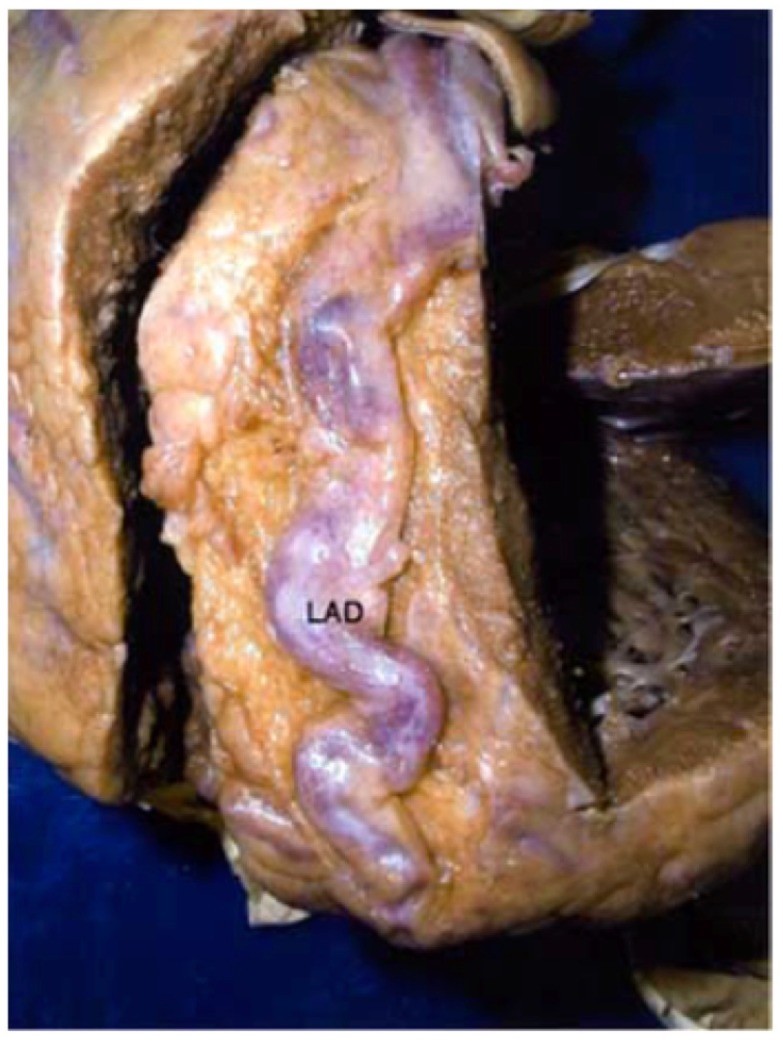
Necropsy specimens showing striking ectasia and tortuosity of the left anterior descending (LAD) coronary artery in a 43 year old female with Eisenmenger syndrome. (From Chugh R, Perloff JK *et al.* Am J Cardiol 2004; 94: 1355-57).

Studies of conduit arteries by Kohler *et al* established a direct relationship between arterial diameter and the viscosity of the perfusate. An increase in flow-mediated shear stress on the luminal surface resulted in elaboration of nitric oxide and prostaglandins which are vasodilators [9]. In CCHD [2] and at high altitude, [8] increased shear stress of the viscous erythrocytotic perfusate initiates coronary artery dilatation, but dilatation subsequently far exceeds expectations anticipated by vasodilator substances alone (Fig. **2**). 

**Fig. (3):**
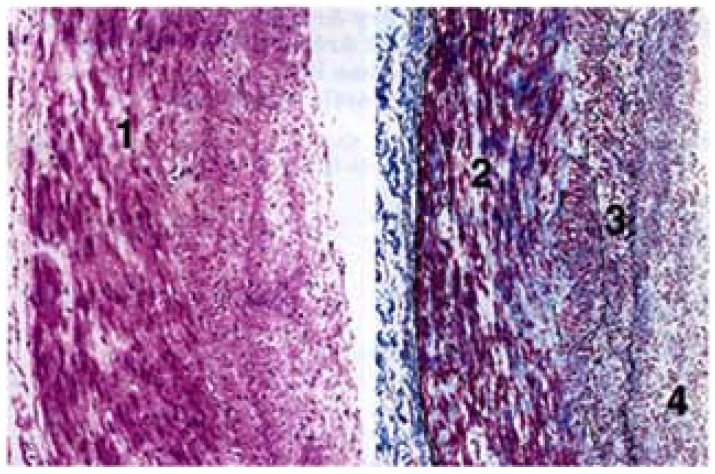
Histology of the ectatic coronary arteries of the patient referred to in (Fig. **2**). 1--loss of medial smooth muscle; 2-- increased medial collagen; 3--duplication of internal elastic lamina; 4--fibromuscular intimal hyperplasia. *Left*, hemotoxylin eosin stain; *right*, Masson’s trichrome stain. (x 200, reduced by 31%). (From Chugh R, Perloff JK *et al.* Am J Cardiol 2004; 94: 1355- 57).

In an attempt to account for the excessive dilatation, necropsy specimens of extramural coronary arteries in adults with CCHD were sectioned and examined histologically at proximal, mid and distal sites along their epicardial courses [2]. At all of these sites, there was loss of medial smooth muscle cells, an increase in medial collagen, an increase in mucopolysaccharides, disruption of internal elastic lamina, and fibromuscular intimal hyperplasia [2] (Fig. **3**). Accordingly, coronary artery dilatation is in response to two variables, namely, elaboration of endothelial vasodilator substances together with morphologic abnormalities that cause medial attenuation [10].

### Basal Coronary Blood Flow and Flow Reserve

Positron emission tomography (PET) was used to determine basal and hyperemic coronary blood flow in the right ventricle, left ventricle and ventricular septum [3]. Basal flow images were first obtained with intravenous N-13 ammonia [3]. After physical decay of the ammonia, dipyridamole was infused as a pharmacologic means of inducing stress. Flow images were then recorded after a second dose of ammonia. 

**Fig. (4):**
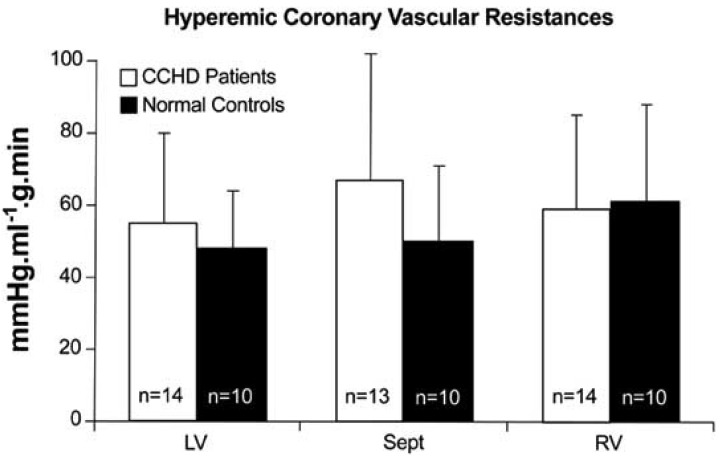
Regional hyperemic coronary vascular resistances were normal in patients with CCHD and in controls. (From Brunken RC, Perloff JK *et al.* Am J Physiol 2005; 289: H1798-806).

Basal flow was appreciably increased in all three regions of interest, but hyperemic flow and flow resistance were comparable to normal controls [3] (Fig. **4**). An increase in basal flow potentially encroaches upon flow reserve, exposing the myocardium to the risk of stress-induced ischemia. 

### The Coronary Microcirculation

**Fig. (5):**
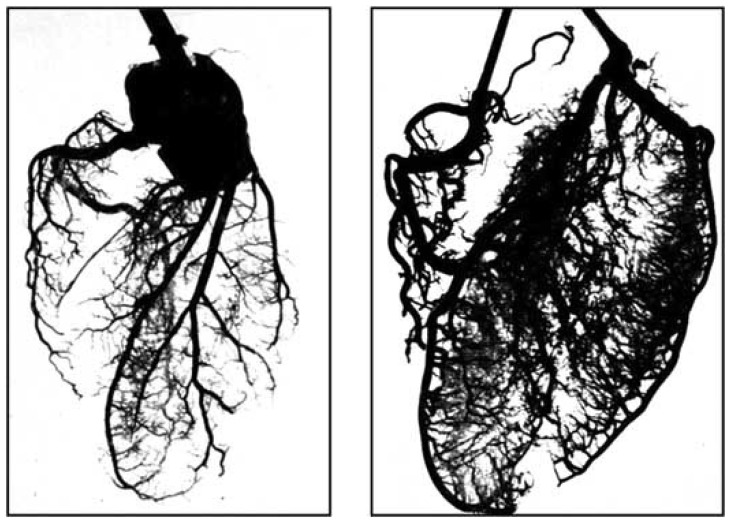
Acrylic resin casts showing normal coronary microcirculation at sea level (left) and striking elaboration of the microcirculation athigh altitude. (From Arias-Stella J, Topilsky M. High Altitude Physiology: Cardiac and Respiratory Aspects. London Churchill- Livingston 1971).

Preservation of hyperemic flow is believed to reside in the coronary microcirculation[3] (Tomanek 2010) rather than in an increase in myocardial oxygen extraction, or in further dilatation of the extramural coronary arteries which are already maximally dilated. In hypoxemic erythrocytotic residents acclimatized to high altitude, acrylic resin casts disclosed coronary angiogenesis and arteriogenesis and a striking increase in the number of peripheral ramifications and secondary branches arising from the main coronary arteries [8] (Fig. **5**).

Based on these observations, it was hypothesized that preservation of hyperemic flow and flow reserve in adults with CCHD resulted from remodeling of the coronary microcirculation. [4] To test this hypothesis, morphometric analyses were done to compare pre-capillary coronary arterioles in CCHD to pre-capillary arterioles in hypertrophied but structurally normal hearts, to hypertrophied but structurally abnormal hearts, and to structurally normal non-hypertrophied hearts [4]. 

Diameter, length, volume and surface densities were determined in coronary arterioles with external diameters of 6mm to 50 mm, and with uninterrupted actin positive smooth muscle outlines. Sections were immuno-labeled with a monoclonal Cy3-conjugated ant zi-smooth muscle actin antibody. Fluorescence images were captured into a computer. In the CCHD group, lower arteriolar density was associated with an increase in diameter, indicating that remodeling upstream from terminal arterioles is a key mechanism for preserving hyperemic flow and flow reserve [4]. Enhanced capacity to vasodilate probably coexists [4].

### Coronary Artery Atherogenesis 

Hypoxemic erythrocytotic residents acclimatized to the high altitude of Cerro de Pasco in the Peruvian Andes are hypocholesterolemic, are virtually devoid of clinically overt coronary artery disease, and were devoid of coronary atherosclerosis in 300 necropsies [8,12]. A study in New Mexico revealed a lower age-adjusted mortality from atherosclerotic heart disease in males living at high altitudes compared to those living at low altitudes [12]. 

Because hypoxemic erythrocytic adults with CCHD might be analogous to hypoxemic erythrocytotic adults residing at high altitude [13], four groups were studied: *Group A*---143 unoperated cyanotic patients aged 18 to 69 years with systemic arterial oxygen saturations of 57% to 73 %; 

*Group B*---47 cyanotic patients rendered acyanotic by surgery performed between ages 22 years and 69 years (mean postoperative follow-up 16.9 years); *Group C*—41 acyanotic unoperated patients aged 22 to 75 years; and *Group D*—48 patients who were acyanotic before and after surgery, mean post-operative follow-up 15 years. [5] No patient had ever taken a cholesterol-lowering medication. All were born and raised at sea level.

**Fig. (6):**
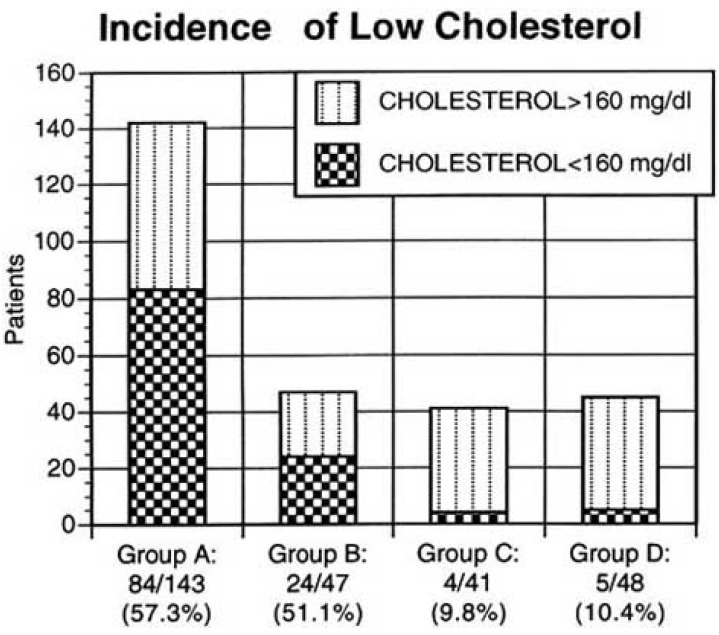
Incidence of low total cholesterol levels (< 160 mg/dl) according to the Framingham norms in Groups A, B, C, and D. (From Fyfe A, Perloff JK *et al.* Am J Cardiol 2005; 96: 283-90).

Fatty streaks and raised lesions appear in the general population at age 15 to 34 years. However, in cyanotic Group A and B patients who ranged in age from the fourth to the sixth decade, there was neither necropsy evidence nor angiographic evidence of coronary atherosclerosis [5]. Cyanotic unoperated Group A patients and cyanotic Groups B patients who were rendered acyanotic by operation had significantly lower total cholesterol levels than acyanotic unoperated or acyanotic operated Group C and D patients (Fig. **6**). 

**Fig. (7):**
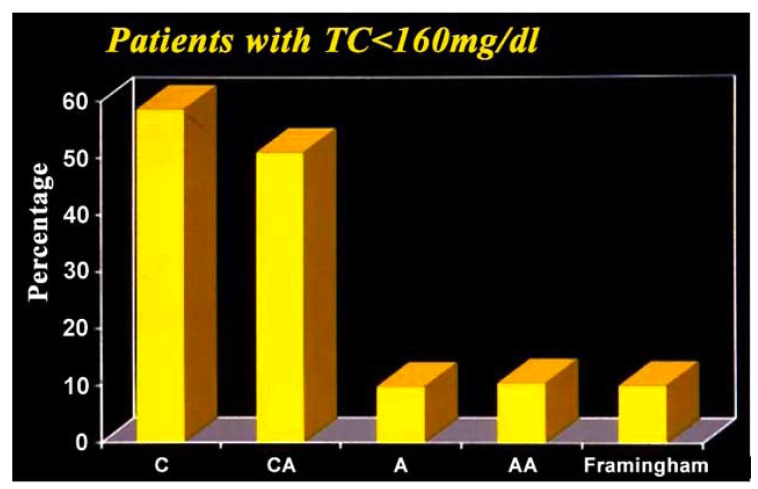
C= Inherently cyanotic unoperated patients. CA— Inherently cyanotic patients rendered acyanotic by operation. A= Inherently acyanotic unoperated patients. AA=Patients inherently acyanotic before and after operation. Low total cholesterol as defined in (Fig. **6**). (From Fyfe A, Perloff JK *et al.* Am J Cardiol 2005; 96: 283-90).

Low levels of total cholesterol defined in the Framing-ham Study as <160 mg/dl, [13] occurred in 58% of cyanotic unoperated patients {Group A}. Low levels persisted after surgical elimination of cyanosis in 52 % of Group B patients (Fig.**7**). Only 11% of cyanotic patients who were hypocholesterolemic before surgery experienced a postoperative rise in total cholesterol levels > 160 mg/dl.

The hypocholesterolemia primarily reflected reductions in LDL cholesterol, with lesser reductions in VLDL. Four variables associated with CCHD are believed to account for hypocholesterolemia: cyanosis, hypoxemia, erythrocytosis, and a genetic predilection. Cyanosis and hypoxemia are obligatory but insufficient causes, and need not be present at birth. This is consistent with observations that within two years after sea level residents ascend to high altitude, their total cholesterol, LDL cholesterol and HDL cholesterol reach the low levels of indigenous high altitude residents [8,12]. It is not known whether high altitude hypocholesterolemia persists after descent to sea level, but in CCHD, hypocholesterolemia does persist after surgical elimination of cyanosis and hypoxemia. 

Hypocholesterolemia has been reported in polycythemia rubra vera which is acyanotic non-hypoxemic erythrocytosis, but in other myeloproliferative disorders, there is no correlation between hematocrit and total cholesterol [14]. Persistence of hypocholesterolemia after surgical elimination of hypoxemia and erythrocytosis implies that once the gene(s) responsible for reduced cholesterol levels are expressed, their effects persist despite elimination of the initiating stimulus [5]. Lack of a relationship between cholesterol levels and age at the time of surgical repair, and failure of cholesterol levels to normalize after surgical elimination of hypoxemia and erythrocytosis, suggest that developmental genetic programming maintains childhood levels and cholesterol profiles, and prevents emergence of adult lipoprotein characteristics. [5] 

In addition to hypocholesterolemia, four co-existing but independent variables contribute to the low incidence of coronary atherosclerosis in CCHD, Fig. (**6**), namely, hypoxemia, upregulation of nitric oxide, hyperbilirubinemia, and low platelet counts [5]. Hypoxemia is associated with a reduction in oxidized plasma LDL and in a reduction of atherogenic intimal oxidized LDL [5]. Nitric oxide, a ubiquitous signaling molecule synthesized from L arginine and oxygen, is upregulated in CCHD because of an increase in endothelial shear stress of the viscous erythrocytotic perfusate which is a major factor in nitric oxide elaboration and eNOS gene expression [15]. Nitric oxide is an anti-atherogenic molecule because it counters platelet aggregation, stimulates disaggregation of preformed platelet aggregates, inhibits monocyte adherence and infiltration, and turns off transcription of intercellular adhesion molecule-1 which governs the endothelial adhesion of monocytes and inhibits smooth muscle proliferation [15]. Unconjugated bilirubin is an endogenous antioxidant that inhibits LDL oxidation and reduces atherosclerotic risk [16]. Gilbert’s syndrome, a benign disorder of bilirubin metabolism, is accompanied by elevated levels of unconjugated bilirubin and immunity from coronary atherosclerosis [16]. Bilirubin is formed from the breakdown of heme, a process that is excessive in CCHD because an increase in red cell mass necessarily coincides with an increase in uncongugated bilirubin. [17]. Low platelet counts are anti-atherogenic [18], and platelet counts in CCHD are typically low or thrombocytopenic. 

## CONCLUSIONS 

Extramural coronary arteries in CCHD initially dilate in response endothelial nitric oxide and prostaglandins which are elaborated because of increased shear stress of the viscous erythrocytotic perfusate. Aneurysmal dilatation—coronary ectasia—results from mural attenuation caused by coexisting abnormalities of the media. Basal coronary blood flow is substantially increased in the dilated extramural coronary arteries, but flow reserve and hyperemic flow remain normal because the coronary microcirculation is remodeled by vascular endothelial growth factor (vasculogenesis) and nitric oxide (angiogenesis). Dilated extramural coronary arteries are atheroma-free because of the anti-atherogenic effects of hypocholesterolemia, hypoxemia, up-regulation of nitric oxide, hyperbilirubinemia, and low platelet counts. The conclusions in this report are not believed to reflect a change in prevalence [19.20] or profile of congenital heart disease [21]. 

## DISCLOSURE

This manuscript is a revised, extended and updated version of my previously published manuscript.
